# Impact of palliative chemotherapy and best supportive care on overall survival and length of hospitalization in patients with incurable Cancer: a 4-year single institution experience in Japan

**DOI:** 10.1186/s12904-019-0428-3

**Published:** 2019-06-03

**Authors:** Yasuko Murakawa, Masato Sakayori, Kazunori Otsuka

**Affiliations:** Department of Medical Oncology, Miyagi Cancer Center, Nodayama 47-1, Medeshima, Natori, Japan

**Keywords:** Best supportive care, Hospitalization, Palliative chemotherapy, Performance status, Quality of life

## Abstract

**Background:**

This study aimed to analyze the determinants of patients’ choice between palliative chemotherapy and best supportive care (BSC) and to investigate how this choice affects overall survival (OS) and length of hospitalization according to Eastern Cooperative Oncology Group (ECOG) performance status (PS).

**Methods:**

An oncologist explained the palliative chemotherapy and BSC options to 129 patients with incurable cancer during their first consultation. Data on the ECOG PS, treatment decision, OS, and the length of hospitalization were retrospectively collected over 4 years.

**Results:**

Patients with an ECOG PS of 0–2 chose palliative chemotherapy more often than those with an ECOG PS of 3–4 (*P* < 0.01). Patients with ≤70 years chose palliative chemotherapy more often than those with > 70 (*P* < 0.05). And patients with gastric cancer and colon cancer chose palliative chemotherapy more often than those with CUP (carcinoma of unknown primary) (*P* < 0.05, *P* < 0.05 respectively). Factors associated with a significantly poorer OS in an adjusted analysis included the ECOG PS and treatment decision (hazard ratios: 0.18 and 0.43; *P* < 0.001, *P* < 0.01 respectively). In patients with an ECOG PS of 0–2, palliative chemotherapy was not associated with a longer OS compared with BSC (median OS: 14.5 vs. 6.8 months, respectively; *P* = 0.144). In patients with an ECOG PS of 3–4, palliative chemotherapy resulted in a significant survival gain compared to with BSC (median OS: 3.8 vs. 1.4 months, respectively; *P* < 0.05). Strong positive correlations between OS and the length of hospitalization were observed in patients with an ECOG PS of 3–4 who underwent palliative chemotherapy (*r*^2^ = 0.683) and the length of hospitalization was approximately one-third of their OS.

**Conclusions:**

The determinants for treatment choice were age, ECOG PS and type of cancer, not sex difference. Oncologists should explain to patients that OS and the length of hospitalization vary according to the ECOG PS when selecting between palliative chemotherapy and BSC.

**Electronic supplementary material:**

The online version of this article (10.1186/s12904-019-0428-3) contains supplementary material, which is available to authorized users.

## Background

In advanced cancer, the therapeutic goal of oncologists is not to achieve a cure, but rather to control symptoms, prevent complications, prolong survival, and maintain as high a quality of life (QOL) as possible [1]. A shorter length of hospitalization is preferable for the QOL of all patients and their families [2, 3]. Hospital-administrated chemotherapy is perceived to be more distressing than chemotherapy at home [4]. However, looking at the Japanese healthcare system from an international perspective, the average length of hospitalization is extremely long [5].

For patients with incurable cancer, best supportive care (BSC) not including palliative chemotherapy may be an important option in some cases [6]. Several studies have shown that palliative chemotherapy generally does not prolong survival in patients with a poor Eastern Cooperative Oncology Group (ECOG) performance status [7, 8]. The American Society of Clinical Oncology advocates withholding palliative chemotherapy in patients with solid tumors and an ECOG PS of 3–4 and recommends BSC instead [9, 10].

The approach to palliative chemotherapy differs considerably depending on the expertise and perspectives of the physician, and oncologists tend to select aggressive chemotherapy [11–14]. BSC is typically not recommended by oncologists if other treatment options are available, including phase I clinical trials [15]. For patients, BSC is often perceived as a negative choice (as “doing nothing”) [16]. Hence, patients typically prefer palliative chemotherapy based on the potential to live longer rather than maintaining QOL [17].

This study aimed to analyze the determinants of patients’ choice between palliative chemotherapy and BSC and to investigate how this choice affects overall survival (OS) and length of hospitalization according to the ECOG PS.

## Methods

In the present study, we retrospectively evaluated 129 patients with incurable cancer who attended the Miyagi Cancer Center (Natori, Japan). Patients with specific malignancies (e.g., metastatic breast cancer or blood malignancies) who had the potential to achieve a significantly longer OS with palliative chemotherapy than with BSC were excluded from this study. Palliative radiation was not an option in the patients of this study.

The oncologist explained the benefits and limitations of palliative chemotherapy and BSC to all patients du ring their first consultation. The emphatic points of the explanation of the treatment options were as follows: (1) The aim of palliative chemotherapy is not to achieve a cure, (2) the adverse effects of palliative chemotherapy may reduce QOL and lead to hospitalization, (3) it is possible to have BSC whenever necessary, (4) all patients eventually have only BSC, (5) BSC can be administered at home, and (6) patients receive full support for symptom relief.

The present study collected data on the ECOG PS and treatment decision (palliative chemotherapy vs. BSC) between May 2013 and May 2014 and on the OS and length of hospitalization between May 2013 and May 2017.

### Statistical analyses

Differences in treatment decisions according to age (≤70 years vs. > 70 years), sex, type of cancer, and ≤ ECOG PS (0–2 vs. 3–4) were evaluated using logistic regression analysis. A multivariate Cox regression analysis was performed to adjust for confounding factors of OS (age, sex, type of cancer, ECOG PS, and treatment decision). A two-tailed *P* value of < 0.05 was considered significant. OS curves were estimated using the Kaplan-Meier method and compared using the log-rank test. The correlation between OS and the length of hospitalization was examined using scatter plot analysis. Coefficient of determination: r^2^ ≥ 0.5 was considered strong correlation, 0.5 > r^2^ ≥ 0.1 was considered moderate correlation. All statistical analyses were performed using Statistical Package for the Social Sciences for Windows (software version 24; SPSS Inc., Chicago, IL, USA).

## Results

As shown in Table 1, a total of 129 patients with gastric cancer, colon cancer, esophageal cancer, miscellaneous malignant tumors (pancreatic cancer, sarcoma, bile duct cancer, duodenal cancer, ureteral cancer, bladder cancer, and anal cancer), and carcinoma of unknown primary (CUP) were enrolled. The majority of patients had a good ECOG PS (ECOG PS 0–2 [*n* = 108] and ECOG PS 3–4 [*n* = 21]). In this study, 101(78.3%) patients had palliative chemotherapy and 28 (21.7%) patients had BSC. More patients with CUP preferred BSC compared with those with gastric cancer and colon cancer (palliative chemotherapy [BSC]: CUP, 1 [6] vs. gastric cancer, 46 [9]; colon cancer, 32 [5], respectively; *P* < 0.05, *P* < 0.05,). More patients aged > 70 years chose BSC compared with those aged ≤70 years (palliative chemotherapy [BSC]: 39 [17] vs. 62 [11], respectively; *P* < 0.05). More patients with an ECOG PS of 0–2 chose palliative chemotherapy compared with those with an ECOG PS of 3–4 (palliative chemotherapy [BSC]: 91 [17] vs. 10 [11], respectively; *P* < 0.01). Sex did not affect treatment decisions (*P* = 0.237). The ECOG PS of patients with CUP was poorer than that of patients with gastric cancer (ECOG PS 0–2 [3, 4]: 2 [5] vs. 49 [6], respectively; *P* < 0.05). [see Additional file 1].

**Table 1:** Multivariate logistic regression analysis of treatment choice in patients with incurable cancer

Variable	Palliative chemotherapy	BSC		OR (95%CI)	*P*-value
	101 (78.3%)	28(21.7%)	129		
Type of cancer					
Gastric ca.	46 (83.6%)	9 (16.4%)	55	18.88 (1.59–224.59)	< 0.05^*^
Colon ca.	32 (86.5%)	5 (13.5%)	37	21.99 (1.74–277.45)	< 0.05^*^
Esophageal ca.	13 (76.5%)	4 (23.5%)	17	13.55 (0.95–192.92)	0.054
MMT	9 (69.2%)	4 (30.8%)	13	5.83 (0.41–83.02)	0.193
CUP	1 (14.3%)	6 (85.7%)	7	1.00 (ref.)	
Sex					
Female	28 (73.7%)	10 (26.3%)	38	0.53 (0.18–1.53)	0.237
Male	73 (80.2%)	18 (19.8%)	91	1.00 (ref.)	
Age(years)					
≤ 70	62 (84.9%)	11 (15.1%)	73	3.32 (1.16–9.54)	< 0.05^*^
> 70	39 (69.6%)	17 (30.4%)	56	1.00 (ref.)	
ECOG PS					
0–2	91 (84.3%)	17 (15.7%)	108	4.99 (1.52–16.42)	< 0.01^*^
3–4	10 (47.6%)	11 (52.4%)	21	1.00 (ref.)	

^*^P < 0.05

Abbreviations: *BSC* best supportive care, *CI* confidence interval, *CUP* carcinoma of unknown primary, *ECOG* Eastern Cooperative Oncology Group, *MMT* miscellaneous malignant tumor, *OR* odds ratio, *PS* performance status, *ref* reference

An ECOG PS of 0–2 was significantly and indepen dently associated with a longer OS compared with an ECOG PS of 3–4 (hazard ratio [HR]: 0.18, 95.0% confidence interval [CI]: 0.10–0.34; *P* < 0.001). Palliative chemotherapy was significantly and independently associated with a longer OS compared with BSC (HR: 0.43, 95.0% CI: 0.24–0.79; *P* < 0.01). Colon and esophageal cancers were associated with a longer OS compared with gastric cancer (HR: 0.49, 95.0% CI: 0.30–0.82; *P* < 0.01 and HR: 0.41, 95.0% CI: 0.20–0.86; *P* < 0.05, respectively). Age, sex, and other types of cancer were not associated with a longer OS (Table 2).

**Table 2:** Multivariate Cox regression analysis

Variable	HR (95.0% CI)	*P*-value
Primary site of the malignancy	–	< 0.05^*^
Colon ca. vs. gastric ca.	0.49 (0.30–0.82)	< 0.01^*^
Esophageal ca. vs. gastric ca.	0.41 (0.20–0.86)	< 0.05^*^
MMT vs. gastric ca.	0.61 (0.29–1.27)	0.186
CUP vs. gastric ca.	0.69 (0.25–1.93)	0.476
Sex (male vs. female)	0.80 (0.50–1.29)	0.367
Age (> 70 vs. ≤70 years)	0.84 (0.54–1.32)	0.455
ECOG PS (0–2 vs. 3–4)	0.18 (0.10–0.34)	< 0.001^*^
Palliative chemotherapy vs. BSC	0.43 (0.24–0.79)	< 0.01^*^

^*^P < 0.05

Abbreviations: *BSC* best supportive care, *CI* confidence interval, *CUP* carcinoma of unknown primary, *ECOG* Eastern Cooperative Oncology Group, *HR* hazard ratio, *MMT* miscellaneous malignant tumor, *PS* performance status

**Fig. 1:**
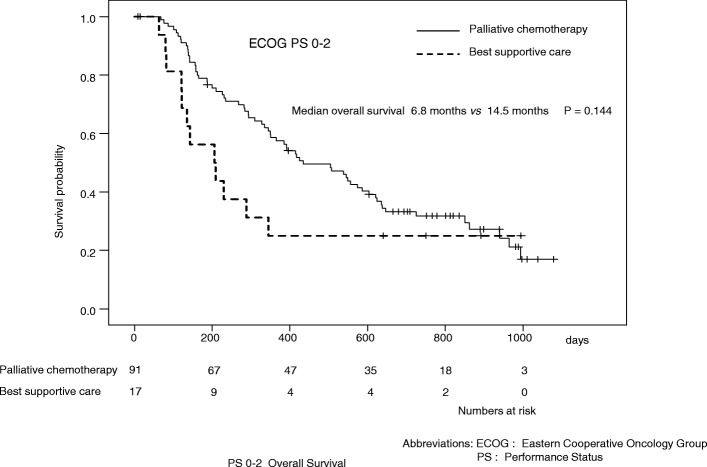
Kaplan-Meier curves of the overall survival of patients with Eastern Cooperative Oncology Group performance status of 0–2

**Fig. 2:**
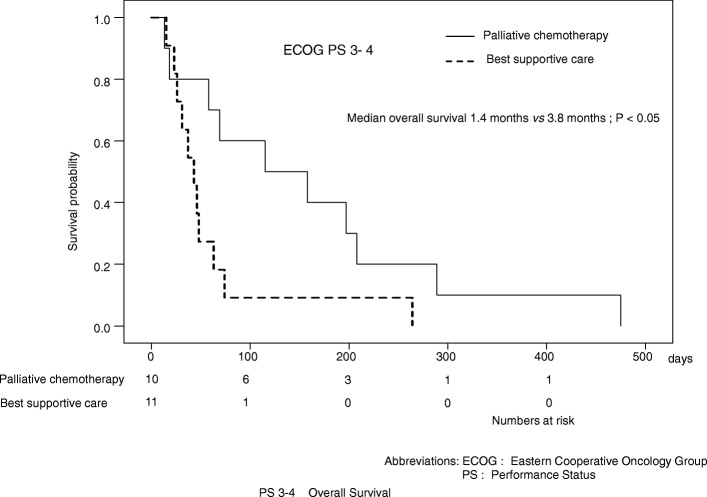
Kaplan-Meier curves of the overall survival of patients with Eastern Cooperative Oncology Group performance status of 3–4

Patients with an ECOG PS of 0–2 who underwent palliative chemotherapy were not associated with a better outcome compared with those who received BSC (median OS: 14.5 vs. 6.8 months, respectively; *P* = 0.144) (Fig. 1). Among patients with an ECOG PS of 3–4, those who received palliative chemotherapy had a better outcome than those who received BSC (median OS: 3.8 vs. 1.4 months, respectively; *P* < 0.05) (Fig. 2). Patients with gastric cancer, ≤70, > 70, and male patients who underwent palliative chemotherapy were associated with a better outcome compared with those who received BSC (2.7 vs. 13.0 months, 1.6 vs. 14.2 months, 4.1 vs. 11.6 months and 2.5 vs. 12.2 months, respectively; *P* < 0.001, *P* < 0.05, *P* < 0.05, *P* < 0.001) [see Additional file 2].

**Fig. 3:**
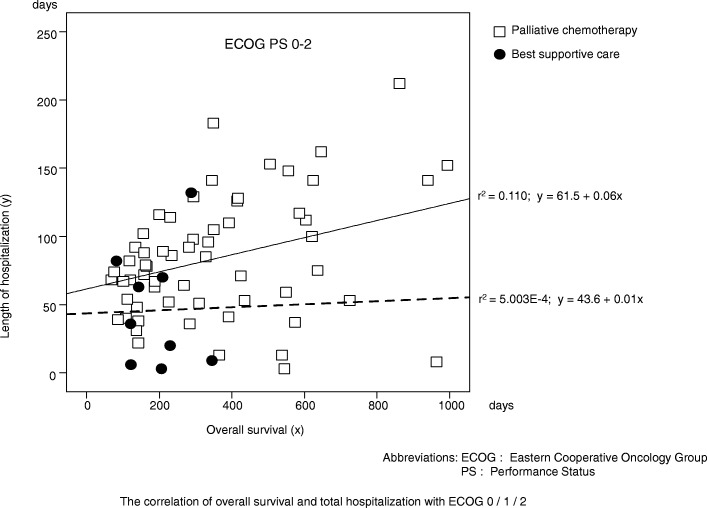
Correlation between overall survival and the length of hospitalization in patients with Eastern Cooperative Oncology Group performance status of 0–2

**Fig. 4:**
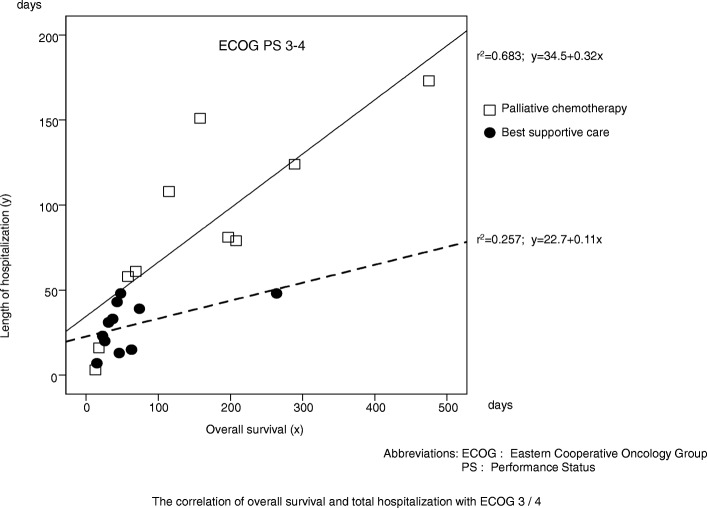
Correlation between overall survival and the length of hospitalization in patients with Eastern Cooperative Oncology Group performance status of 3–4

The correlation between OS and the length of hospitalization was examined using scatter plot analysis in 98 patients who died before Dec. 2018. Among the patients with an ECOG PS of 0–2, a moderate correlation was observed between OS (x-axis) and the length of hospitalization (y-axis) (coefficient of determination: r^2^ = 0.110, y = 61.5 + 0.06x) in palliative chemotherapy, and no correlation in BSC (r^2 =^ 5.003E-4, *y* = 43.6 + 0.01x) (Fig. 3). Conversely, we observed significant positive correlations among the patients with an ECOG PS of 3–4 who had undergone palliative chemotherapy (r^2^ = 0.683, y = 34.5 + 0.32x) and a moderate correlation among the patients with an ECOG PS of 3–4 who had undergone BSC (r^2^ = 0.257, *y* = 22.7 + 0.11x) (Fig. 4). The correlations between OS and the length of hospitalization were also examined in patients with type of cancer, sex and age. The number of patients without gastric cancer were insufficient for the analysis. Among the patients with gastric cancer, ≤70 years and male patients, the moderate correlations were observed in palliative chemotherapy and BSC. Among the patients with > 70 years, the moderate correlation was observed in BSC [see Additional file 3].

## Discussion

The major strength of this study is that an oncologist has provided an in-depth explanation of the treatment options (palliative chemotherapy and BSC) to all patients with incurable cancer during their first consultation, precluding any individual preferences for treatment that the oncologist may have had. The oncologist described the median OS of palliative chemotherapy and BSC to all patients and explained the differences between the two treatment options. The determinants for treatment choice were age and ECOG PS. Specifically, older patients and those with poor ECOG PS had the tendency to undergo BSC. Sex difference and disease without carcinoma of unknown primary had no influence on treatment choice. The tendency of having poor ECOG PS might influence the choice of BSC in patients with carcinoma of unknown primary.

In total, 84% of patients with an ECOG PS of 0–2 chose palliative chemotherapy. The remaining 16% chose BSC because they were either concerned about the adverse effects of chemotherapy or had something that they wanted to accomplish. Only one patient with an ECOG PS of 0–2 who chose BSC changed their mind and later received oral anticancer chemotherapy. Almost half (48%) of the patients with an ECOG PS of 3–4 chose palliative chemotherapy.

Several studies [18, 19] have shown that patients with incurable cancer who received palliative chemotherapy tend to have a longer OS and improved QOL compared with those who received BSC. However, in this study, patients with an ECOG PS of 0–2 did not exhibit a significantly longer OS when they selected palliative chemotherapy over BSC. This may be explained by the fact that some cancers in patients with a good ECOG PS are slow-growing with BSC. Patients with an ECOG PS of 0–2 who chose BSC attended the hospital almost monthly but were not admitted until their general condition worsened. These patients did not experience a reduction in QOL at the beginning of their clinical course. Although patients with an ECOG PS of 3–4 who received palliative chemotherapy had a longer OS than those who received BSC, the increment of survival was small. The degrees of the prolongation of OS by palliative chemotherapy are varied by type of cancer, sex and age.

In this study, the correlation between OS and the length of hospitalization was moderate in patients who received palliative chemotherapy with an ECOG PS of 0–2. However, extension in the length of hospitalization was not also so compared with the extended degree of the OS. Conversely, patients with an ECOG PS of 3–4 exhibited a significant correlation between OS and the length of hospitalization for the patients with palliative chemotherapy. The length of hospitalization in patients with an ECOG PS of 3–4 who received palliative chemotherapy was approximately one-third of their OS. The extension in the length of hospitalization were not also so compared with the extended degree of the OS in patients with male, ≤70 years, > 70 years and gastric cancer who received palliative chemotherapy or BSC.

In principle, care for patients with advanced cancer should include an individual assessment of the patient’s condition and their requirements for treatment throughout the course of their illness. Age is the most important factor for oncologists in deciding whether to recommend palliative chemotherapy or BSC, followed by the patient’s wishes, the length of expected survival, and other factors. When making decisions-making in about cancer treatment, the oncologist should collaborate with the patient and their family members to reach a shared decision [20, 21]. A lower preference for participating in decision-making in patients with incurable cancer was shown to be associated with a stronger preference for palliative chemotherapy [22]. Several studies [23–25] have suggested that oncologists should explain end-of-life care, including BSC, to their patients to reduce aggressive care and increase patient satisfaction.

However, complications can arise in shared decision-making. Patient treatment decisions are influenced based on whether the oncologist emphasizes the positive or negative aspects of the treatment (e.g., survival gain or the probability of dying) [26]. Few oncologists explain to patients how OS can be prolonged in detail as this is often difficult to predict [27, 28]. It is also difficult for oncologists to propose BSC to their patients as it could be perceived as “bad news” [29]. One study reported that only 30% of patients received an explanation about BSC from their oncologists [30]. Shared decision-making is also difficult for patients with incurable cancer [31]. The cognitive function and judgment abilities of these patients typically decline due to aging. Only approximately 60% of patients with incurable cancer understood the purpose of palliative chemotherapy [32]. Therefore, it is often the oncologist who decides the treatment [33, 34].

Treatment for advanced cancer also varies depending on the healthcare environment/system and culture. Several studies have reported on the types of treatment that patients with incurable cancer receive, including intensive palliative chemotherapy and BSC [35, 36]. In Japan, patients with incurable cancer often receive aggressive treatment, including palliative chemotherapy, until the end of life [37].

It is also understood from a previous study that provision of palliative chemotherapy toward the end of life is associated with frequent hospital admissions and high cost [38].

The originality of this study was that we investigated how the choice between palliative chemotherapy and BSC in patients with incurable cancer at the beginning of their treatment affected the length of hospitalization according to the ECOG PS, although it was a single-institution experience.

This study has several limitations. We conducted a retrospective study as it was difficult to perform a randomized controlled trial of patients with incurable cancer who underwent palliative chemotherapy or BSC at the beginning of the treatment. In this study, determinants of patients’ choice we examined were only 4 factors, age, sex, cancer type and ECOG, therefore, more factors are needed for example, economic conditions, caregivers and the level of cognitive impairment. Typically, QOL is evaluated using a questionnaire. The most widely used measures of cancer-specific health-related QOL are the European Organization for the Research and Treatment of Cancer Quality of Life Questionnaire Core 30 [39] and Functional Assessment of Cancer Therapy-General [40]. However, it is difficult to administer a questionnaire, particularly in patients with a poor ECOG PS, because of their physical and/or mental condition. Hospitalization is regarded as one predictor of QOL [41], therefore, we used the length of hospitalization as a surrogate marker of QOL in this study. An objective evaluation of the QOL of patients with incurable cancer other than the length of hospitalization is needed in future investigations.

## Conclusion

The determinants for treatment choice were age and ECOG PS and not sex difference. Oncologists should be required to explain how the choice between palliative chemotherapy and BSC affects OS and the length of hospitalization in patients with incurable cancer to achieve effective shared decision-making.

## Additional files

Additional file 1: Multivariate logistic regression analysis of ECOG PS in patients with incurable cancer (DOCX 17 kb)

Additional file 2: Median overall survival (comparison between BSC and Palliative chemotherapy) (DOCX 18 kb)

Additional file 3: The correlation between OS and length of hospitalization (DOCX 18 kb)

## Data Availability

The raw data of this study is shared in Figshare. (10.6084/m9.figshare.7782566)

## Abbreviations

BSC: Best supportive care
CUP: Carcinoma of unknown primary
ECOG: Eastern Cooperative Oncology Group
OS: Overall survival
PS: Performance status
QOL: Quality of life
