# Supplementary material for: Impact of palliative chemotherapy and best supportive care on overall survival and length of hospitalization in patients with incurable Cancer: a 4-year single institution experience in Japan
Source: BMC Palliat Care. 2019 Jun 3;18:45. doi: 10.1186/s12904-019-0428-3 (PMC6547558; doi:10.1186/s12904-019-0428-3)
Supplement: Supplementary file 1 — Multivariate logistic regression analysis of ECOG PS in patients with incurable cancer (DOCX 17 kb) [file 12904_2019_428_MOESM1_ESM.docx]

| Variable | ECOG PS 0-2 | ECOG 3-4 |  | OR　(95% CI) | *P*-value |
| --- | --- | --- | --- | --- | --- |
|  | 108 (83.7%) | 21 (16.3%) | 129 |  |  |
| Type of cancer |  |  |  |  |  |
| Gastric ca. | 49 (89.1%) | 6 (10.9%) | 55 | 0.11 (0.015-0.806) | <0.05^*^ |
| Colon ca. | 32 (86.5%) | 5 (13.5%) | 37 | 0.14 (0.018-1.131) | <0.07 |
| Esophageal ca. | 14 (82.4%) | 3 (17.6%) | 17 | 0.15 (0.016-1.396) | 0.10 |
| MMT | 11 (84.6%) | 2 (15.4%) | 13 | 0.13 (0.012-1.447) | 0.10 |
| CUP | 2 (28.6%) | 5 (71.4%) | 7 | 1.00 (ref.) |  |
| Sex |  |  |  |  |  |
| Female | 34 (89.5%) | 4 (10.5%) | 38 | 0.39 (0.099-1.526) | 0.18 |
| Male | 74 (81.3%) | 17 (18.7%) | 91 | 1.00 (ref.) |  |
| Age(years) |  |  |  |  |  |
| ≤70 | 61 (83.6%) | 12 (16.4%) | 73 | 1.64 (0.518-5.193) | <0.40 |
| >70 | 47 (83.9%) | 9 (16.1%) | 56 | 1.00 (ref.) |  |
| Treatment choice | |  |  |  |  |
| Palliative chemotherapy | 91 (90.1%) | 10 (9.9%) | 101 | 0.20 (0.060-0.655) | <0.01^*^ |
| BSC | 17 (60.7%) | 11 (39.3%) | 28 | 1.00 (ref.) |  |

Additional file 1 Multivariate logistic regression analysis of ECOG PS in patients with incurable cancer

^*^*P* < 0.05

Abbreviations: BSC, best supportive care; CI, confidence interval; CUP, carcinoma of unknown primary; ECOG, Eastern Cooperative Oncology Group; MMT, miscellaneous malignant tumor; OR, odds ratio; PS, performance status; ref., reference.
