# Supplementary material for: Impact of palliative chemotherapy and best supportive care on overall survival and length of hospitalization in patients with incurable Cancer: a 4-year single institution experience in Japan
Source: BMC Palliat Care. 2019 Jun 3;18:45. doi: 10.1186/s12904-019-0428-3 (PMC6547558; doi:10.1186/s12904-019-0428-3)
Supplement: Supplementary file 2 — Median overall survival (comparison between BSC and Palliative chemotherapy) (DOCX 18 kb) [file 12904_2019_428_MOESM2_ESM.docx]

|  | n |  | n | Median overall survival (months) | p-value |
| --- | --- | --- | --- | --- | --- |
| Type of cancer | | | | | |
| Gastric ca. | 55 | BSC | 9 | 2.7±0.8 | <0.001^*^ |
|  |  | Palliative chemotherapy | 46 | 13.0±1.8 |  |
| Colon ca. | 37 | BSC | 5 | 9.6±8.8 | 0.659 |
|  |  | Palliative chemotherapy | 32 | 19.5±6.3 |  |
| Esophageal ca. | 17 | BSC | 4 | 2.5±3.5 | 0.273 |
|  |  | Palliative chemotherapy | 13 | 28.7±13.1 |  |
| MMT | 13 | BSC | 4 | 7.0±0 | 0.457 |
|  |  | Palliative chemotherapy | 9 | 5.3±1.0 |  |
| CUP | 7 | BSC | 6 | 1.4±1.8 | 0.758 |
|  |  | Palliative chemotherapy | 1 | 2.3 |  |
| Sex | | | | | |
| Female | 38 | BSC | 10 | 6.9±2.0 | 0.527 |
|  |  | Palliative chemotherapy | 28 | 16.8±4.1 |  |
| Male | 91 | BSC | 18 | 2.5±0.6 | <0.001^*^ |
|  |  | Palliative chemotherapy | 73 | 12.2±1.6 |  |
| Age | | | | | |
| ≦70 | 73 | BSC | 11 | 1.6±1.0 | <0.05^*^ |
|  |  | Palliative chemotherapy | 62 | 14.2±2.6 |  |
| >70 | 56 | BSC | 17 | 4.1±1.3 | <0.05^*^ |
|  |  | Palliative chemotherapy | 39 | 11.6±1.9 |  |

Additional file 2 Median overall survival (comparison between BSC and Palliative chemotherapy)

^*^*P* < 0.05

Abbreviations: BSC, best supportive care; CUP, carcinoma of unknown primary; MMT, miscellaneous malignant tumor
