# Supplementary material for: Impact of palliative chemotherapy and best supportive care on overall survival and length of hospitalization in patients with incurable Cancer: a 4-year single institution experience in Japan
Source: BMC Palliat Care. 2019 Jun 3;18:45. doi: 10.1186/s12904-019-0428-3 (PMC6547558; doi:10.1186/s12904-019-0428-3)
Supplement: Supplementary file 3 — The correlation between OS and length of hospitalization (DOCX 18 kb) [file 12904_2019_428_MOESM3_ESM.docx]

Additional file 3 The correlation between OS and length of hospitalization

| Characteristic | n |  | n | r2 |  |
| --- | --- | --- | --- | --- | --- |
| Type of cancer |  |  |  |  |  |
| Gastric ca. | 48 | BSC | 9 | 0.138 | y=45.3₋0.09x |
|  |  | Palliative chemotherapy | 39 | 0.141 | y=54.9+0.07x |
| Sex |  |  |  |  |  |
| Female | 29 | BSC | 7 | 0.023 | y=15.5+0.03x |
|  |  | Palliative chemotherapy | 22 | 0.086 | y=61.5+0.05x |
| Male | 69 | BSC | 16 | 0.295 | y=26.0+0.18x |
|  |  | Palliative chemotherapy | 53 | 0.172 | y=58.3+0.09x |
| Age |  |  |  |  |  |
| ≤70 | 54 | BSC | 8 | 0.466 | y=27.1+0.25x |
|  |  | Palliative chemotherapy | 46 | 0.350 | y=54.3+0.11x |
| ＞70 | 44 | BSC | 15 | 0.114 | y=15.6+0.10x |
|  |  | Palliative chemotherapy | 29 | 0.022 | y=81.1₋0.03x |

Abbreviations: BSC, best supportive care
